# Risk Stratification of Endometrial Cancer Patients: FIGO Stage, Biomarkers and Molecular Classification

**DOI:** 10.3390/cancers13225848

**Published:** 2021-11-22

**Authors:** Jenneke C. Kasius, Johanna M. A. Pijnenborg, Kristina Lindemann, David Forsse, Judith van Zwol, Gunnar B. Kristensen, Camilla Krakstad, Henrica M. J. Werner, Frédéric Amant

**Affiliations:** 1 Department of Obstetrics & Gynaecology, Amsterdam University Medical Centres, 1105 AZ Amsterdam, The Netherlands; J.c.kasius@amsterdamumc.nl (J.C.K.); Judith.vanzwol@amsterdamumc.nl (J.v.Z.); 2 Department of Obstetrics & Gynaecology, Radboudumc, 6500 HB Nijmegen, The Netherlands; hanny.ma.pijnenborg@radboudumc.nl; 3 Department of Gynaecologic Oncology, Oslo University Hospital, 0188 Oslo, Norway; klinde@ous-hf.no; 4 Institute of Clinical Medicine, University of Oslo, 0318 Oslo, Norway; 5 Department of Gynaecology and Obstetrics, Haukeland University Hospital, 5021 Bergen, Norway; david.forsse@uib.no (D.F.); camilla.krakstad@uib.no (C.K.); 6 Institute for Cancer Genetics and Informatics, Department of Oncology, Division of Cancer Medicine, Oslo University Hospital, 0424 Oslo, Norway; gbk@ous-hf.no; 7 Department of Obstetrics and Gynaecology, GROW, Maastricht University School for Oncology & Developmental Biology, 6202 AZ Maastricht, The Netherlands; erica.werner@mumc.nl; 8 Department of Oncology, KU Leuven, 3000 Leuven, Belgium; 9 Department of Gynaecology, Netherlands Cancer Institute, 1066 CX Amsterdam, The Netherlands

**Keywords:** endometrial cancer, uterine neoplasm, surgical staging, FIGO stage, molecular classification, TCGA, ProMisE, TRANSPORTEC

## Abstract

**Simple Summary:**

Endometrial cancer (EC) is the most common gynaecologic malignancy in developed countries. Most patients are sufficiently treated with removal of uterus, tubes and ovaries. It depends on the estimated risk of metastases at diagnosis if more extensive surgery (removal of lymph nodes, peritoneum and/or omentum), to detect small metastases, is indicated. Metastases are associated with a higher risk of recurrence and justify adjuvant treatment (i.e., radiotherapy and/or chemotherapy). Recently it is advised to also subdivide EC into four molecular subgroups. Each subgroup is highly associated to a certain risk of recurrence and helps to decide for adjuvant treatment. What surgery should be performed in each of the subgroups is currently unknown. Moreover, it is uncertain if integration of other factors into the molecular classification could help to improve the risk classification. This review summarizes different aspects of surgery. Moreover, the relation between metastases and other factors including molecular classification are evaluated.

**Abstract:**

Endometrial cancer (EC) is the most common gynaecologic malignancy in developed countries. The main challenge in EC management is to correctly estimate the risk of metastases at diagnosis and the risk to develop recurrences in the future. Risk stratification determines the need for surgical staging and adjuvant treatment. Detection of occult, microscopic metastases upstages patients, provides important prognostic information and guides adjuvant treatment. The molecular classification subdivides EC into four prognostic subgroups: POLE ultramutated; mismatch repair deficient (MMRd); nonspecific molecular profile (NSMP); and *TP53* mutated (p53abn). How surgical staging should be adjusted based on preoperative molecular profiling is currently unknown. Moreover, little is known whether and how other known prognostic biomarkers affect prognosis prediction independent of or in addition to these molecular subgroups. This review summarizes the factors incorporated in surgical staging (i.e., peritoneal washing, lymph node dissection, omentectomy and peritoneal biopsies), and its impact on prognosis and adjuvant treatment decisions in an era of molecular classification of EC. Moreover, the relation between FIGO stage and molecular classification is evaluated including the current gaps in knowledge and future perspectives.

## 1. Introduction

Endometrial cancer (EC) is the most common gynaecologic malignancy in developed countries and its prevalence is increasing [1]. Although the overall survival (OS) of EC patients is known to be relatively good, still 10–15% of patients with low-risk EC develop a recurrence, whereas about 50% of patients with high-risk EC do not recur [2,3,4,5] (Table 1). One of the biggest challenges in EC treatment has been the preoperative risk stratification. Correct estimation of the risk of metastases at diagnosis and the risk of recurrence in the future enables the best-tailored cancer management, will improve prognosis and reduce side effects of unnecessary adjuvant treatment.

Traditionally, the Federation of Gynaecology and Obstetrics (FIGO) staging system aims to provide guidance on treatment and prognosis. Originally, the FIGO staging system was a clinical system, but in 1988 a surgically-based system was adopted, considering the degree of local, regional and distant tumour spread [6,7]. Complete surgical staging involves: total hysterectomy, bilateral salpingo-oophorectomy (BSO), pelvic and para-aortic lymph node dissection. Omentectomy, peritoneal biopsies and peritoneal washings are less routinely performed and considered mostly in non-endometrioid EC (NEEC) patients. Detection of occult, microscopic metastases upstages patients, provides important prognostic information and may guide adjuvant treatment.

The molecular classification of EC by The Cancer Genome Atlas (TCGA) has demonstrated the prognostic relevance of four subgroups: *POLE* ultramutated; mismatch repair deficient (MMRd)/hypermutated; nonspecific molecular profile or copy number low (NSMP/p53wt); and copy number high/*TP53* mutated (p53abn) [5]. This classification was developed making use of postoperative tumour specimens. How surgical staging should be adjusted based on preoperative molecular profiling is currently unknown. Moreover, little is known whether and how other known prognostic biomarkers affect prognosis prediction independent of or in addition to these molecular subgroups.

This review aims to summarize the rationale behind surgical staging and its impact on prognosis and adjuvant treatment decisions in an era of molecular classification of EC. Moreover, the relation between FIGO stage and molecular classification is evaluated including the current gaps in knowledge and future perspectives.

## 2. Factors Incorporated in Surgical Staging

### 2.1. Cytology

EC cells obtained from peritoneal washings, i.e., positive cytology, was defined as FIGO stage IIIA in 1988. Since then, surgical staging of EC routinely included evaluation of peritoneal cytology, even though several publications questioned the prognostic value of positive peritoneal cytology [8,9]. The incidence of positive peritoneal cytology in otherwise low-risk patients without other evidence of extra-uterine disease was found to be variable and overall low, <10%. Moreover, positive cytology as solitary risk factor was reported to have no impact on survival [8]. Patients with FIGO stage I and FIGO stage IIIA based on positive cytology, had comparable overall 5-year survival rates of >90% [9]. As a result, peritoneal washings were omitted from the staging system in 2009. Nonetheless, due to conflicting results in large retrospective studies, the debate on the significance of positive peritoneal washings is ongoing [10,11,12,13]. Specifically in NEEC histology, positive cytology does seem to affect survival. Han et al. reported a disease-free survival DFS of 120 months for patients with negative washings versus 22 months with positive washings (*p* < 0.01) [14].

### 2.2. Peritoneal Biopsies

Peritoneal spread in EC is classified as FIGO stage IVB. The method of peritoneal assessment varies and is not commented upon in the European guideline [4]. Minimally required is a thorough inspection of the peritoneum and sampling of suspicious lesions during surgery. The available evidence on the value of random peritoneal samples in absence of peritoneal abnormalities, as is recommended in ovarian cancer, is limited [15]. Recently, the presence of peritoneal metastases in EC was evaluated in a large retrospective Dutch cohort including 42,333 patients [16]. In only 2% micro- and/or macroscopic peritoneal metastases were present, and found to be associated with higher grade, serous or clear cell histology, lymph node metastasis (LNM) and other distant metastases. Although peritoneal metastases are more frequent in NEEC, literature specifically in this population is sparse, yet in clear cell and serous EC microscopic peritoneal disease in clinically stage I patients was reported in up to 25% [17,18]. The prognostic impact of isolated, occult peritoneal metastases has not been evaluated, likely due to the fact that it is such an uncommon finding. Pragmatically, peritoneal sampling could be considered in NEEC patients as the prognostic impact of peritoneal spread is high. However, more research is needed to evaluate if routine peritoneal sampling is of any clinical value. 

### 2.3. Omentectomy

Omental metastases upstage EC patients to FIGO IVB. There is consensus about omentectomy in case of macroscopic disease and as part of surgical staging in patients at high risk of omental spread, i.e., patients with serous, undifferentiated histology or carcinosarcoma [4]. The prevalence of omental metastasis in clinical stage I was 8.2% in a published meta-analysis: 4.4% for endometrioid EC (EEC) and 9.8% for NEEC [19]. Omental metastases were microscopic in 26.5% and were associated with other sites of disease such as LNM, adnexal or appendix involvement. The number of patients with solely microscopic omental metastases was not reported, which is important to consider in case omentectomy merely serves a diagnostic purpose. Based on the analysis of a national database of 9097 patients with high-grade EC, omentectomy did not affect survival (hazard ratio: 0.94, 95% confidence intervals: 0.84, 1.05) [20]. However, since this database was not designed to answer this question, important details on the pre-operative work-up, intra-operative findings, extent of the surgery, histology and decision making in surgical and adjuvant treatment could not be assessed. Other publications do report a positive effect of omentectomy in NEEC on survival supporting even a therapeutic role. Ross et al. suggests a prognostic role alone, as omental sampling in patients with uterine carcinosarcoma did not significantly influence survival, though the presence of omental metastases did, 11.4 versus 128.7 months, respectively (*p* < 0.001) [21]. Evidence for omentectomy in other histologic types, such as clear cell, is conflicting [4,19,21]. Overall, omentectomy improves the quality of staging and hence is predictive of prognosis. This information allows to decide on the need for adjuvant treatment and hence helps to avoid side effects of unnecessary treatment.

### 2.4. Lymphadenectomy

In 1970 the Gynaecologic Oncology Group (GOG) performed a pilot trial investigating positive para-aortic and pelvic lymph nodes during hysterectomy and BSO. Respectively, 11% and 10% of the patients with presumed FIGO stage I had LNM [3]. Subsequently, systematic pelvic and para-aortic lymphadenectomy was introduced as part of the surgical staging procedure. Since 1988, spread to pelvic or para-aortic lymph nodes has been classified as FIGO stage IIIC, and was refined in 2009 by a subdivision into pelvic (IIIC1) and para-aortic (IIIC2) LNM. Currently, lymphadenectomy is recommended in high-risk EC patients as lymph node status determines FIGO stage and adjuvant treatment recommendations, yet debate about its therapeutic benefits in presumed low- and high-risk patients is ongoing [4,22].

The best available evidence about routine lymphadenectomy is based on results of two randomized controlled trials (RCTs) published by Benedetto Panici et al. in 2008 and Kitchener, et al. in 2009 [23,24]. Pelvic lymphadenectomy was compared to no lymphadenectomy in a cohort of in total 1851 EC patients. No differences were found in DFS or OS between the two groups [22,23,24]. Subgroup analysis of all classified disease stages and possible interfering risk factors found no statistic difference in OS and recurrence-free survival (RFS) [24]. Critics state that the lack of therapeutic effect of lymphadenectomy is due to study limitations as none of the RCTs systematically included para-aortic lymph nodes, the number of lymph nodes removed was suboptimal, and lymph node status did not influence adjuvant therapy strategies [25,26].

Recently, a meta-analysis was published comparing pelvic lymphadenectomy to pelvic and para-aortic lymphadenectomy [27]. Thirteen retrospective cohort studies including 7349 patients were identified. Patients with combined pelvic and para-aortic lymphadenectomy compared to pelvic lymphadenectomy had a better 5-year OS of 85% vs. 76% (relative risk (RR) 1.13, 95% CI 1.04–0.24, I2 = 57.3%). Subgroup analysis in a formerly published meta-analysis indicated that the positive effect of combined pelvic and para-aortic lymphadenectomy could only be detected in intermediate- or high-risk patients [28]. No significant difference in OS was found in low-risk EC patients, defined as FIGO stage IA, grade 1–2 and endometrioid histology without lymphovascular space invasion (LVSI). Unfortunately, the quality of evidence is low as retrospective cohort studies are accompanied by a high risk of bias. Whether or not systematic sampling of para-aortic lymph nodes could influence the therapeutic value of lymph node dissection remains unclear and is currently evaluated (ClinicalTrials.gov, NCT03438474, 10 November, 2021). 

The number of lymph nodes removed during lymph node dissection is known to be relevant. Cragun et al. reported a significantly improved survival in patients who had >11 pelvic lymph nodes removed during lymph node dissection [29]. In patients with high-grade EC with removal of >11 vs. <11 pelvic lymph nodes the 5-year OS was 88% and 79% respectively (hazard ratio (HR) 0.25; *p* < 0.0001). Benedetto Panici et al. had >11 lymph nodes removed in 11% and Kitchener, et al. had >10 lymph nodes removed in 65% of the patients in their lymphadenectomy groups [23,24]. Subgroup analysis determining the effect of the number of lymph nodes removed could not be performed in detail. Therefore, also the importance of systematic lymphadenectomy, defined as removal of >11 pelvic lymph nodes, remains a topic of debate. 

Most importantly, the unanswered question remains if the results of the two RCTs on the impact of lymph node dissection in EC patients would have been different had lymph node status influenced adjuvant treatment. In that case, lymphadenectomy could have an indirect positive effect on the prognosis.

### 2.5. Sentinel Lymph Node Procedure

Sentinel lymph node biopsy (SLN) represents an approach to reduce staging-associated morbidity while maintaining accuracy. SLN provides excellent sensitivity and negative predictive value for LNM and is accepted as a staging procedure equivalent to lymphadenectomy [4]. For optimal performance the method depends on surgeon experience, following a strict algorithm (including ipsilateral lymphadenectomy in the case of failed mapping) and pathological ultrastaging [4]. In a recent meta-analysis comprising seven studies, pooled results supported significantly lower incidences of lower leg lymphedema and post-operative complications for SLN compared to lymphadenectomy [30]. Prospective evidence regarding survival effects and patient-reported outcomes is not yet available. A drawback of routine SLN compared to (imaging-based) selective lymphadenectomy may be the need for centralization and increase in health care costs. As the majority of clinical stage I patients do not have LNM, patients in whom staging can safely be omitted after preoperative triage may be treated by a general gynaecologist. SLN should be performed at centres with sufficient surgical volume. In conclusion, the accuracy of sentinel lymph node biopsy to detect LNM also in patients with high-grade EC facilitates surgical staging with limited surgery-related morbidity [31].

## 3. FIGO Stage: An Important Prognostic Factor Guiding Adjuvant Treatment

### 3.1. Traditional Important Prognostic Factors

**Figure 1:**
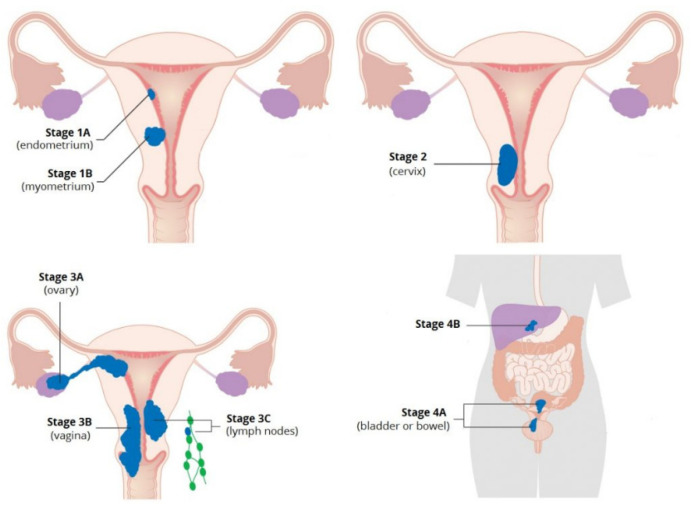
Surgically based FIGO staging system for endometrial cancer. Adapted from Cancer Research UK [CC-BY-SA-4.0] [32].

The FIGO staging system represents a structured overview of relevant prognostic factors, incrementally leading to a higher stage. The system has evolved over time as prognostic data accumulated. It indicates survival and allows treatment comparison (Figure 1). Due to this background, the relation between FIGO stage and survival is unequivocal. The Danish Gynecologic Cancer Group (www.DGCG.dk, 15 September 2021) nicely illustrates this by providing population-based reports using the FIGO staging system. In the years 2005–2017, the 5-year survival was 85.1%, 72.2%, 47.0% and 15.6% for stages I–IV, respectively. The survival rates increased to 86.3%, 74.6%, 48,7% and 28.2% for the years 2016–2020. 

Histological type and grading are other important traditional prognostic factors to consider. Their classification is defined by the WHO Classification of Tumours of Female Reproductive Organs. The prognostic importance of tumour cell invasion into lymph-vascular spaces (LVSI) has been recognized since the last revision of the FIGO guidelines and is implemented in the most recent international guidelines on EC treatment [4,33,34]. Using FIGO stage, histologic type, grade and LVSI status, and the classification rules defined by ESGO-ESTRO-ESP 2020 a good stratification into risk groups can be obtained [35].

### 3.2. Relevance of Full Surgical Staging in Presumed Early Stage, Low Risk Patients

The majority of EC patients present with presumed low-risk disease, signifying endometrioid grade 1–2 histology on a curettage or endometrial biopsy and disease confined to the uterus. Available adjuvant treatment modalities such as radiotherapy and/or chemotherapy serve to eliminate or reduce residual disease after surgery but increase the length and cost of treatment and most importantly increase the risk of morbidity at short- and long term [36,37,38]. When the disease is limited to the uterus, a total hysterectomy and BSO is currently performed and associated with minimal iatrogenic morbidity. The risk of occult metastatic spread, predominantly to regional lymph nodes, is, however, significant at around 10% and heavily impacts prognosis as previously discussed. Surgical staging thus serves to identify patients where treatment directed to extra-uterine disease is indicated, and to avoid overtreating the majority of patients with localized disease. Confirmed nodal status serves to tailor treatment. Irrespective of the preferred institutional approach to adjuvant radiotherapy, it seems evident from available data that adjuvant chemotherapy should be considered for patients with node-positive disease [37,38], whereas the role of chemotherapy in node-negative high-risk disease is more unclear, as seen, for example, in GOG249 [39]. This data supports the need of surgical staging. Further refinement of stratification is likely important for this group of patients.

Several preoperative algorithms have been proposed to identify in which patients a staging lymphadenectomy can safely be omitted, i.e., selective lymphadenectomy. The Mayo criteria were early out to gain widespread acceptance and suggested < 50% myometrial invasion, grade 1–2 endometrioid histology and size < 2 cm as criteria for treating with hysterectomy only, identifying a population with 5% LNM and 5-year disease specific survival (DSS) at 97% [40]. The Mayo criteria have been criticized for being overly conservative, leading to lymphadenectomy in approximately 2/3 of patients, and have been modified several times. The Korean gynaecological oncology group has suggested an algorithm based on preoperative pelvic MRI and preoperative circulating level of Cancer Antigen 125 (CA125), which identifies 40–50% of patients as low-risk with a false negative rate of 1.3%, and has been validated in different cohorts [41]. The ongoing search for biomarkers that identify LNM in presumed early-stage patients has resulted in numerous potential candidates, but no preoperative marker is yet widely in use. The ongoing MoMaTEC2 (Molecular Markers in the Treatment of Endometrial Cancer 2) is assessing the implementation of hormone receptor expression in a preoperative algorithm to reduce lymphadenectomy rates (ClinicalTrials.gov, NCT02543710, 10 November 2021). Another approach is to group several biomarkers based on various techniques into one instrument, the ENDORISK algorithm by Reijnen, et al. is one such example. It was developed within the ENITEC network, using a Bayesian network incorporating both clinical and immunohistochemical biomarkers. External validation showed an area under the curve (AUC) of 0.82 (95% CI 0.76–0.88) for LNM and 0.82 (95% CI 0.77–0.87) for 5-year DSS. Over 50% were classified as low risk with <5% risk of LNM, and a false-negative rate of 1.6% [42]. The addition of advanced imaging modalities has despite great hopes not led to any breakthrough as of yet and is limited by costs and availability. 18F-FDG-Positron Emission Tomography combined with Computed Tomography (CT) (PET/CT) has better sensitivity and specificity for detection of lymph node metastases than CT and Magnetic Resonance Imaging (MRI) [43,44], but still fails to identify small lesions where the FDG-uptake is not above background levels. Thus, selective lymphadenectomy strategies can reduce the rate of patients that undergo lymphadenectomy at the cost of a limited number of false negative stage III patients. 

### 3.3. Relevance of Full Surgical Staging in Patients with Parametrial, Vaginal or Adnexal Spread

In patients with macroscopic FIGO stage III, debulking surgery is recommended and results in a significantly improved PFS, and OS when complete or optimal cytoreduction can been achieved [45]. As shown in a recent meta-analysis, optimal and complete debulking could be obtained in respectively 69.8% and 82.2% and was not related to tumour histology [46]. Subsequent administered chemotherapy resulted in a 5-year survival rate of 50%, when compared to 23% in patients with neo-adjuvant chemotherapy followed by interval debulking, and 11% with chemotherapy only [47]. As no prospective data are available, the results of these studies might be affected by selection bias. Microscopic stage III on the contrary, can only be recognized after proper surgical staging, and has a better outcome when compared to the macroscopic stage III EC. The PORTEC-3 trial randomized patients with high-risk EC, including patients with stage III (n = 295; 45%), between external beam radiation (EBRT) and chemoradiation (CRT) followed by four courses of platinum-based chemotherapy. The 5-year OS was 78.7% in patients that received chemoradiotherapy compared to 69.8% in those with adjuvant radiotherapy [37]. Unfortunately, subgroup analysis for FIGO IIIA-B and IIIC was not presented. In the GOG-258 study 736 patients with FIGO III/IVA were randomized to chemotherapy only or chemoradiation followed by chemotherapy without a survival benefit for either of the treatment modalities [38]. Subgroup analysis within FIGO III lacked statistical power to discriminate preferred treatment modality in IIIA, IIIB and IIIC1–2. 

Weelden et al. provided specific data for FIGO IIIA–B and C patients within a large population-based study [48]. Subgroup analysis did not observe a benefit of combined treatment modalities in FIGO IIIA–B. The majority of patients with stage IIIA–B EC did not undergo lymphadenectomy. Interestingly, in those who underwent extensive lymphadenectomy (>20 nodes), the outcome was significantly better than that in those without extensive lymphadenectomy. This highlights the relevance of proper staging to determine adjuvant therapy. 

The presence of adnexal involvement in endometrial cancer can be classified as either metastatic, i.e., FIGO IIIA, or synchronous primary endometrial and ovarian tumours. Schultheis, et al. demonstrated by massively parallel sequencing of presumed sporadic synchronous EECs and ovarian carcinomas that these lesions were clonally related [49]. This was confirmed in a series of 50 cases with synchronous endometrial and ovarian carcinomas in which 92% shared at least one somatic mutation [50]. Molecular profiling according the TCGA confirmed prognostic relevance for the four groups. Interestingly, *TP53* mutation and extra-utero-ovarian disease were independent predictors for outcome, underlining the relevance of surgical staging for adjuvant systemic treatment planning.

## 4. Molecular Risk Classification

### 4.1. Origin and Rationale behind the Molecular Risk Classification

From the early publication by Bokhman, EC has been divided in two clinicopathological subtypes; type 1 (EEC) and type 2 (NEEC), in which the type 1 was considered low-grade and oestrogen dependent and type 2 high-grade and non-oestrogen dependent [51,52]. Later, these subtypes were shown to be associated with specific molecular aberrations, such as microsatellite instability (MSI), and mutations in *PTEN, KRAS*, and *CTNNB1* (beta-catenin) mutations in type 1 and *TP53*, *HER2*, and loss of heterogeneity in type 2 [53]. However, even among expert gynaecological pathologists, poor to moderate reproducibility was shown in subtype classification in high-grade EC, especially if diagnosis was based on haematoxylin eosin slides only [54,55,56]. Immunohistochemical analysis, in specific p53 and the oestrogen and progesterone hormone receptors (ER, PR), has for a longer time been applied to support the subtyping in less straightforward cases [57]. Subsequently, it became clear that not all tumours fitted this dichotomous classification. This was especially the case for a number of the high-grade tumours including mixed subtypes, p53-aberrant endometrioid tumours and ER positive NEEC [58].

Traditionally the FIGO grading system for EEC has been architectural, focusing on solid growth and nuclear atypia, resulting in a three-tier system of low-, median- and high-grade [59]. Subjectivity was also noted here with resulting moderate interobserver agreement in grading EC, especially for grade 2 [60,61]. Modifications, including two-tier systems, have been proposed and shown increased intra- and interobserver reproducibility [61,62,63]. 

Finally, there is known to be considerable discrepancy between the preoperative vs. the postoperative classification in both subtyping and grading, as illustrated by a recent review and meta-analysis by Visser, et al. [64]. They concluded there is only moderate agreement on tumour grade between preoperative sampling and final histology, with grade 2 showing the lowest agreement. For subtyping a better agreement was noted between non-endometrioid and especially the endometrioid histology.

The challenging areas indicated above result from the morphological as well as clinical heterogeneity that exemplifies endometrial carcinoma. Therefore, the creation of a molecular classification by the TCGA study was warmly welcomed [5,65].

### 4.2. Overlap between Histology Based Risk Groups and Molecular Risk Groups

The histopathological risk stratification is based on a number of variables of which subtype, grading, myometrial invasion depth, nodal and other metastasis, and more recently (significant) LVSI are among the most relevant [4,66]. Immunohistochemical markers are used to support the risk stratification, mainly by assigning the histological subtype. Among the most frequently used immunohistochemical markers are p53, PTEN, ER and PR, where aberrant p53 expression supports histological subclassification and retained ER and PR receptor status is associated with less aggressive disease [67,68]. In addition, MSI proteins are frequently assessed for Lynch syndrome testing [4]. 

In contrast, the TCGA molecular study applied genomic, transcriptomic and proteomic analysis. Based on a combination of somatic nucleotide substitutions, MSI and somatic copy number analyses (SCNA) they stratified into four defined groups; a *POLE* exonuclease domain mutated group, critical for DNA proofreading and resulting in ultramutated tumours (POLE); a microsatellite instable group, with mutations in the microsatellite genes resulting in hypermutated tumours (MMRd); a group characterized by extensive SCNA and otherwise very frequent *TP53* mutations (p53abn), and a rest group with few group-specific molecular characteristics although mutations in the PI3K/AKT pathway were commonly seen as well as Wnt pathway mutations (NSMP) [5].

To facilitate clinical use, great effort has been put into development of pragmatic substitutes that can be analysed in routinely available formalin fixed paraffin embedded tissue. This resulted in a similar although not identical classification into four groups [69]. MMRd and *TP53* mutations appeared to be equally reliably tested by immunohistochemistry compared to sequencing [5,70,71,72]. Only *POLE* mutation status remains so far dependent on targeted sequencing of the *POLE* exonuclease domain.

Although in both risk stratification methods immunohistochemistry is thus applied for p53 and MSI proteins, it serves a different purpose. Whereas immunohistochemical testing in the classical histologic assessment supports the diagnosis (p53abn) or identifies patients with Lynch syndrome (MMRd), it provides here the assessment of the molecular group and therefore helps in risk stratification [4,73].

**Figure 2:**
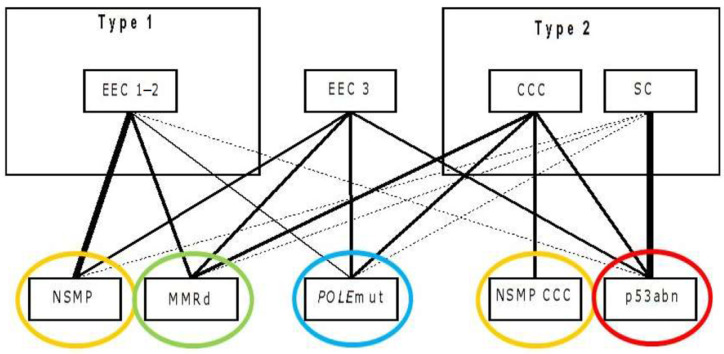
Relation between the traditional histologic classification and the molecular classification. Each traditional histologic diagnosis is connected to the molecular subgroup they represent. The thicker the connecting line, the stronger the relation. The figure demonstrates that each molecular classification is detected in each histologic subgroup, though NSMP is overrepresented amongst grade 1 and 2 EEC and p53abn cancers are most often diagnosed in patients with SC. EEC: endometrioid endometrial cancer, CCC: clear cell carcinoma, SC: serous cancer. NSMP: nonspecific molecular profile, MMRd: mismatch repair deficient, POLE: *POLE* ultramutated, p53abn: copy number high/*TP53* mutated. Modified from UpToDate Endometrial cancer: Pathology and classification by Huvila J, MD, PhD, McAlpine JN, MD, FACOG, FRCPSC, available from: URL: https://www.uptodate.com/contents/endometrial-cancer-pathology-and-classification?source=history_widget (accessed on 10 November 2021).

Despite the fact that all histotypes are represented in all four molecular subgroups, a certain overlap exists [65,74] (Figure 2). The p53abn group predominantly contains serous type ECs, whereas the NSMP group almost entirely consists of endometrioid histotype, grade 1 and 2 EC. The POLE and MMR-D groups mostly contain endometrioid type EC, yet all histotypes and grades are represented. Over half of the clear cell carcinomas is found in the p53abn group and 40% in the NSMP group. Carcinosarcomas mainly fall into the p53abn group and might have a worse prognosis than serous EC in the p53abn group. Whether combining information on histotype with the molecular subgroup could improve prognosis prediction is under study [74,75]. 

## 5. Knowledge Gaps and Possible Solutions

### 5.1. Can Molecular Risk Classification Predict Treatment Response?

The prognostic value of the molecular subgroups is well-established and the recent ESGO/ESTRO/ESP guidelines recommend the integration of molecular classification to tailor adjuvant therapy [4]. Yet, the predictive relevance of molecular subgroups is mainly extracted from retrospective studies, hampering translation in definite treatment algorithms and lacking analyses of relatively rare subgroups. Therefore, the current standard advice for adjuvant treatment is not unequivocal in each molecular subgroup.

*TP53* mutant EC, diagnosed in 26% of EC patients, has the worst prognosis and is thought to be chemotherapy sensitive. The recently published post hoc analysis of patients with available molecular profile included in PORTEC 3 revealed that patients with p53abn tumours derived a larger absolute benefit in 5-year RFS from combined adjuvant chemoradiation compared to radiotherapy alone. Patient numbers in subgroup analysis were, however, small, and the difference in RFS was significant in patients with FIGO I disease, but not in FIGO III, which could be due to the lack of systematic staging in this subgroup [76]. The study was originally not powered for analysis by molecular subgroups and results need to be interpreted with caution due to the exploratory nature of the analysis. 

There may be other biomarkers where more reliable data exist regarding the prediction of response to treatment. In particular among patients with p53abn tumours, HER-2/neu is a promising predictive marker [77]. The addition of trastuzumab to chemotherapy in patients with stage III/IV or recurrent HER-2/neu positive uterine serous cancer led to a significant OS benefit compared to chemotherapy alone [78]. The suggested benefit from the combination of chemotherapy with bevacizumab in patients with *TP53* mutation included in GOG-86P is limited by the lack of a control group and the post hoc retrospective design of this analysis [79]. 

In about 7% of all EC patients a *POLE* mutation is detected [5]. Because of the favourable prognosis in general, no adjuvant treatment is advised in patients with FIGO I or II disease [4]. However, due to its rarity, evidence is still lacking to guide adjuvant treatment in FIGO III or IV disease. 

There is so far insufficient evidence for the role of MMR status for response to radio- or chemotherapy [76,80,81] but in recurrent EC, there is solid evidence that MMR status is predictive of response to immunotherapy [82,83]. Among patients with MMRd tumours, the prognostic significance of Lynch-like versus sporadic MSI EC has recently been reported in patients treated with pembrolizumab indicating that differences in the immune-cell tumour microenvironment play a role in predicting response [84]. 

Patients not classified as p53abn, MMRd or POLE are designated NSMP and account for 39% of all EC patients [85]. Together with the MMRd patients (28%), they are associated with intermediate prognosis and represent the largest molecular subgroup (67%). Further risk stratification in addition to molecular profiling is required to define adjuvant treatment in this subgroup.

Finally, the issue of the ‘multiple classifiers’ in molecular profiling EC needs to be addressed. Luckily, it is reported to be an infrequent finding that an EC specimen can be subdivided into more than one molecular subgroup. Though, it is important to realise that sequential determination of the molecular subgroup according to ProMisE would not detect all ‘multiple classifiers’ [85]. In a group of 3518 ECs, initially 5% appeared to two classifiers of which 3% had another mutation next to the *TP53* mutation [86]. Morphologically, MMRd and p53abn, and POLE and p53abn classifiers had most similarities with MMRd and POLE EC, respectively. Moreover, in these cases, *TP53* mutation seemed to be a secondary event that occurred during tumour progression. Finally, the survival of the ‘multiple classifiers’ was found to be alike the molecular subgroup with the best prognosis. More data is warranted to confirm these results and to find out what applies to other molecular subgroup combinations. 

### 5.2. Can Molecular Risk Classification Guide Surgical Management?

The traditional morphology-driven staging strategy is now challenged since the molecular profiling according TCGA points towards a paradigm shift from morphological to molecular classification in which we currently lack prospective and complete data that link each of the four categories to a metastatic spread pattern [5,85,87,88]. 

Retrospective studies have indeed shown that the p53abn genotype is more frequently diagnosed in high stage disease but information is not available for stage II, III and IV separately. In addition, this data is similarly incomplete for the other categories [5,85,87,88]. Therefore, the molecular classification does not help surgeons to decide whom and how to stage EC. Future studies need to address the metastatic spread pattern of the four different categories thereby guiding EC staging procedures. Until we have this information, surgeons need to rely on the traditional morphology based staging strategy [89].

### 5.3. What Is the Significance of Integrating Molecular Classification, Histopathology, Other Prognostic Biomarkers and FIGO Stage?

There is sufficient data to support that molecular profiling adds a necessary layer to increase diagnostic and prognostic precision in a part of EC patients. However, large gaps remain in the knowledge on how molecular subgroups can help us to predict prognosis or treatment response in each individual patient. Combination of molecular classification and morphology, prognostic biomarkers and/or FIGO stage might offer a solution.

MMRd tumours are often associated with adverse prognostic factors such as high grade and LVSI [80]. Still, patients have better than expected outcomes, actually comparable to patients with MMR proficient tumours. It has been hypothesized that this may be explained by the improved anti-tumour immune response in these patients. In addition in the POLE group, there is a considerable number of patients presenting with high-risk features despite their indolent clinical behaviour. A recent meta-analysis described the histopathologic features and the prevalence of the ESMO risk category for each molecular subgroup and highlights that characteristics such as *POLE* mutation status and MMRd are more important than histologic features in prognostication [90]. However, 17% of POLE EC do recur after a median of 30 months follow-up [91]. The by far largest molecular group is the group of patients with NSMP tumours and almost half of the patients will be classified as such. Most of these patients are classified as of low- to intermediate-risk based on histopathology but the group also includes high-risk patients that may carry a poor prognosis [92]. As this group is the least molecularly and prognostically characterized subgroup, we must assume that prognosis is heavily affected and can be determined by other factors beyond *TP53* mutation status [93]. 

In the last decade several papers have been published on biomarkers in endometrial cancer, mainly prognostic, but also diagnostic and predictive. The only proteins that have been extensively studied are CA125, human epididymis protein 4 (HE4), ER/PR expression, MSI proteins, the tumour suppressors *PTEN* and *TP53*, *CTNNBB1* mutation, the cell adhesion molecules E-cadherin (*CDH*1) and neural cell adhesion molecule L1 (*L1CAM*), c-MET pathway, the proliferation marker protein Ki-67 (KI67), and the Erb-B2 Receptor Tyrosine Kinase 2 (ERBB2) [94,95,96]. Loss of ER/PR has been related to clinicopathological criteria such as high grade, deep myometrial invasion and LNM [67]. The expression of ER/PR was studied in relation to the TCGA groups and although predictive for outcome in the univariate analysis, overruled by the ProMisE subtypes in multivariate analysis [97]. Yet, cut-off values for ER/PR of 5% and 1% were used, that might have underestimated their prognostic value as Weelden, et al. demonstrated the relevance of classification EC based on ER/PR into: high (0–10%), intermediate (20–80%), and low-risk (90–100%) groups [98]. A *CTNNB1* mutation was more frequently detected in grade 1 and 2 EEC, though associated to a worse recurrence free survival. L1CAM expression has been widely studied in EC and shown to be an important prognostic marker with strong relation to LVSI, NEEC and LNM [99]. In addition, this adhesion molecule might serve as a treatment target for anti-L1CAM antibody that is currently under development for clinical use. In relation to the molecular subgroups, L1CAM was strongly related to the p53abn group, but also significantly relevant in the NSMP group [100]. In a systematic review and meta-analysis of Reijnen et al. the diagnostic accuracy of clinical biomarkers were studied for preoperative prediction of LNM in EC [101]. Elevated CA125 and thrombocytosis were the strongest biomarkers for the prediction of LNM with diagnostic accuracy of >0.75. Haematological biomarkers were studied in few other studies that also showed the association of thrombocytosis with reduced median OS most pronounced in serous EC [102]. Simple biomarkers such as haematological, serum CA125 and immunohistochemistry are attractive due to the fact that these are relatively cheap and easily accessible, facilitating clinical implementation with limited costs. Research focusing on if and how these biomarkers can improve or even replace molecular risk classification in certain EC patients is of major relevance.

As up till now the presence and location of metastases (i.e., FIGO stage) has been the most important prognostic factor, it seems obvious to integrate molecular classification and stage. Raffone et al. authored a meta-analysis on the clinical features of each of the molecular subgroups. Five studies including 1622 were used to analyse the subdivision of FIGO stage I among the four subgroups [103]. FIGO stage I was observed most frequent in the POLE group (93.7%) and least frequent in the p53abn group (50.8%). Kommos et al. assessed LNM in each of the molecular subgroups. Again, LNM were more likely to be found in the p53abn patients. None of the POLE patients had LNM. The relation between the molecular subgroups and metastases to other locations (i.e., omentum, peritoneum, and systemic) has not been evaluated so far. If and how integration of stage and molecular classification affects prognosis prediction has not been reported.

## 6. Discussion

This review demonstrates that major steps have been taken to refine risk stratification of EC patients. Correct estimation of the risk of metastases at diagnosis and future recurrence in each patient is of major importance as it currently determines the extent of surgery and need for additional treatment. Over the past decades, the FIGO staging system has been adapted based on factors with true prognostic significance. This is ongoing as new study results continue to shape our knowledge. Moreover, new prognostic biomarkers are continuously discovered, facilitating further subdivision of low- and high-risk EC patients. Finally, distinguishing the four molecular subgroups by the TCGA has led to a paradigm shift and a new, alternative, method of EC risk classification.

The limitations of each stratification method have also been discussed. Some of the items included or excluded in FIGO staging are a topic of debate. Positive peritoneal washings do no longer influence FIGO stage, though results from recent publications suggest it may affect prognosis in certain subtypes. LNM do affect prognosis significantly, though the lymphadenectomy procedure has not yet proven its therapeutic value. The fact that in the available studies, lymph node positivity did not influence the choice in adjuvant treatment may contribute to his observation. This might be clarified by the ongoing clinical ECLAT trial (ClinicalTrials.gov, NCT03438474, 10 November 2021). Extensive research is needed before new biomarkers can and will be used in clinical practice. Discovery, validation and clinical applicability assessment need to take place before their implementation. Most likely combinations of several biomarkers will lead to useful risk stratification methods. Although promising, molecular classification is by itself not the solution. The molecular classification is prognostic but does not take surgical stage in consideration. Its predictive value is under investigation. Most patients are classified as intermediate-risk (NSMP and MMRd) and further stratification is important to tailor surgical and adjuvant treatment. Traditionally, EC treatment is guided by a combination of prognostic factors. We believe treatment and survival of EC patients could further improve by integrating the molecular classification with the currently important prognostic factors such as relevant biomarkers and FIGO stage. 

### 6.1. Future Perspectives

#### 6.1.1. Preoperative Risk Stratification

In the past years, innovations in diagnostic and treatment modalities have altered the medical decision process in direction of a more individualized approach. Predictive algorithms, nomograms, and risk-stratification systems have been developed for EC [41,104,105]. However, only a few rely exclusively on preoperative data, and so far none have been implemented in clinical guidelines [41,106]. Molecular profiling according to the TCGA has demonstrated its prognostic value in EC, and may guide adjuvant treatment planning in the future [5,76]. Whether molecular profiling is beneficial in the preoperative setting remains to be elucidated [89]. The p53abn subgroup has eminently been recognized with the worst outcome and a high risk of LNM, and can easily be identified by preoperative immunohistochemistry [90]. Using an alternative approach, as referred to previously, the ENDORISK Bayesian model, based on integration of both clinical and immunohistochemical biomarkers, is highly discriminative with a false negative rate of 1.6% in patients classified as low risk of LNM [42]. This illustrates that preoperative risk stratification by simple biomarkers is feasible and can contribute to a cost-effective work-up. This is supported by Köbel et al., who stresses the importance of waiting for the results of ongoing clinical trials on the value of molecular classification in the clinical context, and assessments of its cost benefit before implementing molecular subtyping [107]. 

#### 6.1.2. Combination of FIGO Stage and Molecular Subgroup

Randall et al. identified EC as a chemotherapy-sensitive disease [108]. Later, two randomized clinical trials (NSGO-EC-9501/EORTC-55991 and MaNGO ILIADE-III) did not show significant improvement in OS after sequential combination of chemotherapy and radiotherapy in high-risk stage I–III EC, including in NEEC [109]. However, in 46% of patients, lymph node status and thus true stage was unknown. Therefore, it remains to be elucidated which group exactly benefits from adjuvant chemotherapy and if addition of radiotherapy to chemotherapy improves the results. The ongoing ENGOT-EN2-DGCG trial (NCT01244789) aims to shed light into this issue by comparing survival in patients with stage I grade 3 or stage II EEC, or stage I and II NEEC without LNM after randomization to adjuvant chemotherapy with brachytherapy allowed in both arms. This study is closed for recruitment and results are eagerly awaited.

Similar studies for the different new molecular groups are needed [89]. Whereas in the morphologic era positive lymph nodes are the most important prognostic factor, it is currently unknown what the prognostic effect is of upstaging for each molecular type. With this information, future studies can test the benefit of adjuvant systemic treatment for each group. When its predictive effects are confirmed, the mutational profile will help choosing the most appropriate treatment schedule. For example, chemotherapy may be beneficial for p53abn EC and immune checkpoint inhibitors may be effective for MMRd EC. However, the clinical benefit in the adjuvant and metastatic setting for each indication still needs to be proven. A high risk for relapse does not equal the fact that adjuvant treatment is effective. Therefore, prospective properly designed randomized clinical studies in the molecular era are needed to answer these questions. A first step should be to prospectively study cohorts of properly staged EC patients in whom molecular subgroup is determined. 

The ongoing PORTEC 4a [110] and the RAINBO trials program are the first prospective (randomized) trials where molecular profiling is used to tailor adjuvant treatment. The latter explores in one of the planned trials the addition of the PARP inhibitor to adjuvant chemoradiation in patients with p53abd tumours building on data that especially NEEC harbour homologous recombination deficiency [111]. The results of these trials are an important next step to determine how molecular profiling can lead to patient tailored surgical and adjuvant treatment and improve survival. The fact that surgical staging is not mandatory is however a missed chance to make solid conclusions per stage of EC. 

#### 6.1.3. Molecular Staging Making Use of Liquid Biopsies

Tumour material such as cells, nucleic acids, exosomes and proteins may be detected and isolated in blood samples (or other fluids) from patients—a liquid biopsy. During the last two decades this source of information has been explored for diagnostics, treatment and surveillance for different cancer types, exemplified by the approval of a circulating tumour DNA (ctDNA) based EFGR-mutation test being approved by the FDA and EMA for non-small cell lung cancer patients where a regular biopsy is unfeasible [112]. For EC, liquid biopsy remains at early stages of research. The two most popular candidates for clinical implementation, circulating tumour cells (CTC) and ctDNA have been examined in relatively few settings and have not yet been refined enough to contribute to patient treatment. The detection rate of CTCs is limited in EC, due to the fact that the majority of cases are at an early stage, and is estimated to be around 7–20% when an EpCAM-based detection system is used [113,114,115]. CtDNA is shown to be detectable with serous histology, while detection in endometrioid histology seems more challenging [116,117]. As detection rates are more impressive for high-risk histology or advanced disease, the main utility of liquid biopsy in endometrial cancer management is expected to lie in early detection of recurrences and response monitoring. Although there is a possibility that circulating tumour material may complement or replace surgical staging for the assessment of extra-uterine disease in the future, many technical and biological issues remain to be solved [118]. Direct genetic testing and matching to targeted treatment may also be a possibility, for example assessing MMR-status in endometrial cancer recurrences, but so far this has only been achieved sporadically and needs refinement and validation [119]. Future research will clarify if molecular staging will be useful in the diagnostic work-up of EC or even influence treatment decisions.

## 7. Conclusions

Over the years new methods have been developed to stratify EC patients into a low-, intermediate-, or high-risk category. These developments are promising in guiding individualized surgical and adjuvant treatment. Tailored EC treatment prevents under- and overtreatment, that can result in suboptimal survival or unnecessary complications and toxicity. Major progress has been made with the introduction of the molecular classification. However, with implementation of new methods the proven traditional methods, such as surgical staging and certain clinic-pathological biomarkers (i.e., LVSI) should not be ignored. Especially FIGO stage, which, alone, has been the most important prognostic factor up till now. The future lies in combinations of traditional and new stratification methods. Based on the results of ongoing research, the method to accurately assess the risk category in each patient will continuously be refined.

## Figures and Tables

**Table 1:** Prognostic risk groups of endometrial cancer patients according to the ESGO/ESTRO/ESP guidelines [4].

Risk Group	Tumour Characteristics
Low	Stage IA EEC grade 1/2 without substantial LVSI
Intermediate	Stage IB EEC grade 1/2 without substantial LVSI or stage IA EEC grade 3 without substantial LVSI or stage IA NEEC without myometrial invasion
High–intermediate	Stage I EEC with substantial LVSI regardless of grade and depth of invasion or stage IB EEC grade 3 regardless of LVSI status or stage II
High	Stage III–IVA with no residual disease or stage I–IVA NEEC with myometrial invasion, and with no residual disease
Advanced metastatic	Stage III-IVA with residual disease or stage IVB

EEC: endometrioid endometrial cancer; LVSI: lymph-vascular space invasion; NEEC: non-endometrioid endometrial cancer.
